# 2-Methoxyestradiol TPGS Micelles Attenuate Cyclosporine A-Induced Nephrotoxicity in Rats through Inhibition of TGF-β1 and p-ERK1/2 Axis

**DOI:** 10.3390/antiox11081499

**Published:** 2022-07-30

**Authors:** Mohammed W. Al-Rabia, Mohamed A. Alfaleh, Hani Z. Asfour, Waleed S. Alharbi, Mohamed A. El-Moselhy, Nabil A. Alhakamy, Usama A. Fahmy, Osama A. A. Ahmed, Omar Fahmy, Omar M. Rashad, Abdulmohsin J. Alamoudi, Ashraf B. Abdel-Naim

**Affiliations:** 1 Department of Medical Microbiology and Parasitology, Faculty of Medicine, King Abdulaziz University, Jeddah 21589, Saudi Arabia; awalrabia@kau.edu.sa (M.W.A.-R.); hasfour@kau.edu.sa (H.Z.A.); 2 Department of Pharmaceutics, Faculty of Pharmacy, King Abdulaziz University, Jeddah 21589, Saudi Arabia; malfaleh@kau.edu.sa (M.A.A.); wsmalharbi@kau.edu.sa (W.S.A.); nalhakamy@kau.edu.sa (N.A.A.); uahmedkauedu.sa@kau.edu.sa (U.A.F.); oaahmed@kau.edu.sa (O.A.A.A.); 3 Clinical Pharmacy and Pharmacology Department, Ibn Sina National College for Medical Studies, Jeddah 21589, Saudi Arabia; melmoselhy@ibnsina.edu.sa; 4 Department of Pharmacology and Toxicology, Faculty of Pharmacy, Minia University, Minia 61519, Egypt; 5 Center of Excellence for Drug Research and Pharmaceutical Industries, King Abdulaziz University, Jeddah 21589, Saudi Arabia; 6 Mohamed Saeed Tamer Chair for Pharmaceutical Industries, King Abdulaziz University, Jeddah 21589, Saudi Arabia; 7 Department of Urology, Universiti Putra Malaysia, Serdang 43400, Malaysia; omarfahmy.ahmed@upm.edu.my; 8 Department of Pharmaceutical Technology, Faculty of Pharmacy and Biotechnology, German University in Cairo, Cairo 11835, Egypt; omar.amin-aly@guc.edu.eg; 9 Department of Pharmacology and Toxicology, Faculty of Pharmacy, King Abdulaziz University, Jeddah 21589, Saudi Arabia; ajmalamoudi@kau.edu.sa

**Keywords:** cyclosporine A, 2-methoxyestradiol, TPGS, nephrotoxicity, TGF-β1, p-ERK

## Abstract

The immunosuppressant cyclosporine A (CSA) has been linked to serious renal toxic effects. Although 2-methoxyestradiol (2ME) possesses a wide range of pharmacological abilities, it suffers poor bioavailability after oral administration. The purpose of this study was to evaluate the potential of 2ME loaded D-ɑ-tocopheryl polyethylene glycol succinate (TPGS) micelles to prevent CSA-induced nephrotoxicity in rats. A 2ME-TPGS was prepared and showed particle size of 44.3 ± 3.5 nm with good entrapment efficiency and spherical structures. Male Wistar rats were divided into 5 groups, namely: Control, Vehicle, CSA, CSA + 2ME-Raw, and CSA + 2ME-Nano. CSA was injected daily at a SC dose of 20 mg/kg. Both 2ME-Raw and 2ME-Nano were given daily at oral doses of 5 mg/kg. Treatments continued for three successive weeks. 2ME-TPGS exerted significant protective effects against CSA nephrotoxicity. This was evidenced in ameliorating deterioration of renal functions, attenuation of pathological changes in kidney tissues, exerting significant anti-fibrotic, antioxidant, and anti-inflammatory effects together with significant anti-apoptotic effects. Western blot analyses showed both 2ME-Raw and 2ME-Nano significantly inhibited protein expression of TGF-β1 and phospho-ERK (p-ERK). It was observed that 2ME-TPGS, in almost all experiments, exerted superior protective effects as compared with 2ME-Raw. In conclusion, 2ME loaded in a TPGS nanocarrier possesses significant protective activities against CSA-induced kidney injury in rats. This is attributable to 2ME anti-fibrotic, antioxidant, anti-inflammatory, and anti-apoptotic activities which are mediated at least partly by inhibition of TGF-β1/p-ERK axis.

## 1. Introduction

The kidney is a vital organ that conducts a variety of functions, including homeostasis regulation, extracellular environment control, and excretion harmful compounds and metabolites [1]. Therefore, toxic compounds have the potential to adversely affect renal functions and cause nephrotoxicity [2]. Unfortunately, this can occur as a result of the toxic effects of certain medications, i.e., drug-induced nephrotoxicity [3]. This kind of disease is characterized by a loss of renal cells and structural alterations of the nephron. It also involves structural alterations of the tubules, interstitial tissue, glomeruli, and intrarenal arteries and veins [4]. Cyclosporine A (CSA) is the first available calcineurin inhibitor that positively influences organ transplantation processes [5,6]. CSA functions by suppressing interleukin-2 gene transcription and cytoplasmic binding to cyclophilin. Consequently, the CSA-cyclophilin complex reduces signaling of calcium and inhibits calcineurin, the calcium-dependent enzyme that translocates and dephosphorylates cytosolic-activating nuclear factor of T lymphocytes [7,8]. CSA, on the other hand, has been linked to a wide variety of serious renal adverse effects, such as acute and chronic renal toxicity, hypertension, electrolyte abnormalities, and tubular acidosis [9]. It is important to understand that CSA-induced nephrotoxicity develops as two distinct types of kidney damage. Acute CSA nephrotoxicity is a fast incident that does not cause any structural damage and allows the kidney’s function to be restored if CSA levels are reduced or the exposure is discontinued [10]. Chronic CSA nephrotoxicity is marked by permanent and increasing renal interstitial fibrosis, which could lead to chronic or irreversible kidney impairment [11,12]. Kidney histopathological changes induced by CSA include tubulointerstitial fibrosis and arteriolar vasculopathy [13]. Mechanisms of nephrotoxicity have been suggested to involve release of vasoactive substances, such as angiotensin II [14], and up-regulation of transforming growth factor-beta (TGF-β) and collagen I and IV [15].

2-Methoxyestradiol (2ME) is an endogenous metabolite of estradiol but with no estrogenic activity [16]. It has antiproliferative [17], antioxidant [18], and anti-inflammatory properties [19]. It possesses pleotropic pharmacological actions. These include anticancer [20], antihypertensive [21,22], vascular-protective [23], and neuroprotective [24] activities. 2-Methoxyestradiol was shown to prevent kidney damage and improves survival in septic mice via inhibiting the production of cytokines and nitric oxide [25]. Numerous research studies have pointed to the possibility that 2ME could be employed as a therapy for renal dysfunction [26,27,28]. Additional studies have reported the inhibiting effect of 2ME on Ang II-induced hypertension and renal impairment in animal models and suggested that 2ME could be beneficial in the treatment of cases of kidney dysfunction and end-organ damage [29]. Clinical trials demonstrated that 2ME has an excellent safety profile with no intolerable side effects [30,31,32]. However, pharmacokinetic data indicates that 2ME has limited bioavailability [33,34] as it is subject to extensive hepatic and extrahepatic metabolism [35]. This restricts its therapeutic value and raises the need for new formulations to enhance its solubility and bioavailability. In this regard, nanotechnology provides a new platform for delivering drugs to treat different types of diseases including nephropathologies [36,37]. In particular, it can enhance renal targeting, retention, and localization [38,39].

D-ɑ-tocopheryl polyethylene glycol succinate (TPGS) is a derivative of Vitamin E. It has attracted researchers’ attention for its ability to self-assemble into nanometric colloidal dispersions of hydrophobic drugs [40,41]. TPGS is an FDA-approved safe adjuvant that is used in contemporary drug delivery systems for its improved solubility, stabilization, permeation of drugs, biocompatibility, and TPGS ability to inhibit P-glycoprotein activity [42,43]. The micellar structure is formed above the critical micellar concentration (CMC) of TPGS [44]. Entrapped drugs within the core provide an architecture that is suitable for controlled release properties, where the hydrophilic shell provides stability for the three-dimensional spherical structure of the system [41,44]. Therefore, the purpose of this study was to evaluate the possible preventive effects of 2ME loading in TPGS micellar structure on CSA-induced nephrotoxicity in rats.

## 2. Materials and Methods

### 2.1. Chemicals

2-Methoxyestradiol (2ME, >98%) was purchased from Fraken Biochem Co., Ltd. (Qingdao, China). Cyclosporine A (CSA) and TPGS were obtained from Sigma-Aldrich (St. Louis, MO, USA). Remaining chemicals were of the highest commercial purity available.

### 2.2. Animals

Adult male Wistar rats of body weight of 200–230 g were obtained from the animal facility, Faculty of Pharmacy, King Abdulaziz University. Animals were kept at a temperature of 22 ± 3 °C with relative humidity of 60–70% and on a standard 12-h light/dark. Animals had free access to food and water. All experimental procedures and animal care in this study were permitted by the Research Ethics Committee, Faculty of Pharmacy, King Abdulaziz University (Reference # PH-1443-63).

### 2.3. Preparation of 2ME TPGS Micelles

The preparation 2ME-loaded TPGS micelles was carried out following the previous report [45]. Briefly, TPGS (500 mg) and 2ME (10 mg) were dissolved stirring in 30 mL ethanol. Using a rotary evaporator, the alcoholic solution was evaporated at 40 °C. After that, the formed 2ME TPGS thin film was dispersed, under shaking, in 50 mL Milli-Q water for 4 h. The aqueous dispersion was then passed through 0.22 µm filter to get rid of un-entrapped 2ME. Plain (vehicle) micelles formula was prepared with the same method without 2ME. Lyophilization was then carried out for the TPGS-based micellar dispersion using a freeze dryer (Martin Christ Gefriertrocknungsanlagen GmbH, Osterode am Harz, Germany).

### 2.4. Characterization of 2ME TPGS Micelles

#### 2.4.1. Particle Size

Particle size of 2ME TPGS micellar dispersion was determined by a light scattering technique using a Nano ZSP particle size analyzer (Malvern, UK). The sample dispersion was diluted with Milli-Q water before measurement where the average of five determinations was recorded.

#### 2.4.2. Entrapment Efficiency %

2ME content in the prepared TPGS-based micelles was calculated using Equation (1) that was analyzed by HPLC after dilution in methanol, as previously described [46].

(1) $$2\mathrm{ME}\;\mathrm{Entrapment}\;\mathrm{Efficiency}\;\%=\frac{2\mathrm{ME}\;\mathrm{amount}\;\mathrm{in}\;\mathrm{the}\;\mathrm{filtrate}}{2\mathrm{ME}\;\mathrm{amount}\;\mathrm{originally}\;\mathrm{added}}\times 100$$

##### 2ME TPGS Micelles Investigation by Transmission Electron Microscopy

2ME TPGS preparation micelles was investigated by a transmission electron microscope (TEM) JEOL GEM-1010 (JEOL Ltd., Tokyo, Japan) at Mycology and Biotechnology Center, Al-Azhar University, Nasr City, Cairo, Egypt. The sample was stained with 1% phosphotungstic acid and visualized by TEM after drying.

### 2.5. Experimental Design

To assess the effects of 2ME nanoparticles on CSA-induced kidney injury, animals were divided randomly into five experimental groups (*n* = 6), and treated as follows: (1) Control group (Normal saline 10 mL/kg/day orally + Normal saline 5 mL/kg/day SC); (2) Vehicle group, i.e., plain TPGS (Vehicle 10 mL/kg/day orally + 5% DMSO in normal saline 5 mL/kg/day SC); (3) CSA group (Normal saline 10 mL/kg/day orally + CSA 20 mg/kg/day SC); (4) 2ME-Raw group (2ME-Raw 5 mg/kg/day orally + CSA 20 mg/kg/day SC); (5) 2ME-TPGS group (2ME-TPGS 5 mg/kg/day orally + CSA 20 mg/kg/day SC). Raw 2ME was dissolved in 5% DMSO in normal saline. Oral doses were given 1 h before SC injections. All treatments continued for six weeks. Chosen doses and regimen are based on a pilot experiment and consistent with dose range published data [23,47,48,49]. Twenty-four hours after the last treatment, animals were anesthetized with IP injection of 50 mg/kg ketamine and 5 mg/kg xylazine and blood samples were collected from the retro-orbital plexus. Blood samples were centrifuged for 10 min at 3000 rpm and sera were collected and stored at −80 °C to be used to determine standard biochemical parameters for kidney function. Then, rats were sacrificed by decapitation, and kidneys were dissected out, rinsed with ice-cooled saline, and sliced into different parts. Part of the kidney tissues were then kept in either 10% formalin or RNAProtect Tissue Reagent (Cat. # 76106, Qiagen, MD, USA). The remaining tissues were kept at −80 °C after flash-freezing in liquid nitrogen for subsequent analysis.

### 2.6. Measurements of Renal Functional Markers

Serum level of creatinine, urea, and cystatin C were determined using commercial kits (Cat. No. E4370-100, BioVision, Milpitas, CA, USA; Cat. No. ab83362, Abcam, Cambridge, UK, and Cat. No. CSB-E08385r, CUSABIO, Fannin, TX, USA, respectively). The BCA Protein Assay Kit (Cat. No. ab102536, Abcam, Cambridge, UK) was used to determine protein concentration.

### 2.7. Histopathology and Immunohistochemistry

Neutral formalin (10%) was used to fix kidney tissues before paraffinization was carried out. Tissues were then cut into 5 µm sections that were stained with either hematoxylin and eosin (H&E), Masson’s trichrome (MTC), Picrosirius red (PSR), or Periodic acid–Schiff (PAS) to investigate renal tubulointerstitial injury induced by CSA. A seasoned pathologist carried out histological examination without knowing treatment groups. Scoring of sections on the basis of abundance within a range from – to +++ was done considering tubular necrosis, tubular degeneration, tubular dilatation, thickened basement membrane, and interstitial fibrosis. For immunohistochemistry, xylene and descending dilutions of ethanol were used to deparaffinize and rehydrate the tissue sections, respectively. For antigen retrieval, the tissue sections were placed in boiling citrate buffer (0.1 M at pH 6.0) for 10 min followed by an incubation in 5% bovine serum albumin (BSA) in tris-buffered saline (TBS) for 2 h. Sections were then stained with Mouse and Rabbit Specific HRP/DAB Detection Kit (Abcam, Cambridge, UK) involving endogenous peroxidase inhibition and utilizing the following primary antibodies against: IL-6 (ab9324), TNF-α (ab205587), COX-2 (ab179800), or NF-κB p65 (ab194726) obtained from Abcam, Cambridge, UK. The sections were then examined, photographed, and quantified blindly. Image analysis was performed using software (ImageJ, 1.8.0_¬112, NIH, Bethesda, MD, USA). Image size was adjusted to 640 × 480 with 8 bit. After calibration Optical Density of fixed representative areas (OD) values were determined in gray scale.

### 2.8. Oxidative Status Markers

Assessment of total protein, malondialdehyde (MDA), superoxide dismutase (SOD), and catalase (CAT) were done with commercial kits (catalog # 10009055, 701780, 706002 and 703002, correspondingly, Cayman^®^ Chemical, Ann Arbor, MI, USA).

### 2.9. Real-Time Polymerase Chain Reaction

Total RNA from kidney tissues was isolated using TRIzol and served as a template in the synthesis of cDNA using Omniscript RT kit (Cat. No. 205113, Qiagen, MD, USA). The assessment of mRNA expression was carried out using the SYBR Green PCR kit (Cat. No. 180830, Qiagen, MD, USA) and validated forward and reverse primers in our labs [50]. β-Actin, Bax, and Bcl2 forward and reverse primers sequences are shown in Table 1. Data were normalized to β-actin and analyzed following the 2^–ΔΔCT^ method [51].

### 2.10. Western Blot

Content of specific proteins in kidney tissues was assessed using Western blotting as described previously [52]. Briefly, ice-cold RIPA lysis buffer containing Phosphatase Inhibitor Cocktail (Catalog # P0001, Sigma-Aldrich, St. Louis, MO, USA) was used to lysate the tissues before determining protein concentration of supernatant using BCA protein assay kit (Biovision Inc., CA, USA). Proteins samples were loaded on 10% sodium dodecyl sulfate-polyacrylamide gels. Following electrophoresis, proteins were transferred to PVDF membranes (Bio-Rad Laboratories, Hercules, CA, USA). This was followed by blocking the membranes using 5% milk in TBS containing 0.1% Tween 20 (TBST) for 1 h before overnight incubation at 4 °C with the following primary antibodies: anti-TGF-β1, anti-p-ERK1/2, and anti-β-actin antibodies (Cat. #. ab215715, ab201015 and ab8226, Abcam, Cambridge, UK). After washing the membranes with TBST, they were incubated for 1 h at room temperature with horseradish peroxide-conjugated secondary antibodies (Cat. No. ab205718, Abcam, Cambridge, UK). This was followed by washing with TBS. Membrane visualization and protein semi-quantification were achieved using ChemiDoc MP Imaging System with Image Lab Software (Bio-Rad Laboratories, Dubai, United Arab Emirates) and ImageJ (ImageJ, 1.8.0_¬112, NIH, MD, USA), respectively.

### 2.11. Statistical Analyses

All results are expressed as means ± SD, and GraphPad Prism (Prism 8.1, GraphPad Software, San Diego, CA, USA) was used for statistical testing by one-way ANOVA followed by Tukey’s multiple comparison test. Statistical significance threshold was set at *p* < 0.05.

## 3. Results

### 3.1. Preparation and Characterization of 2ME-TPGS Micelles

**Figure 1:**
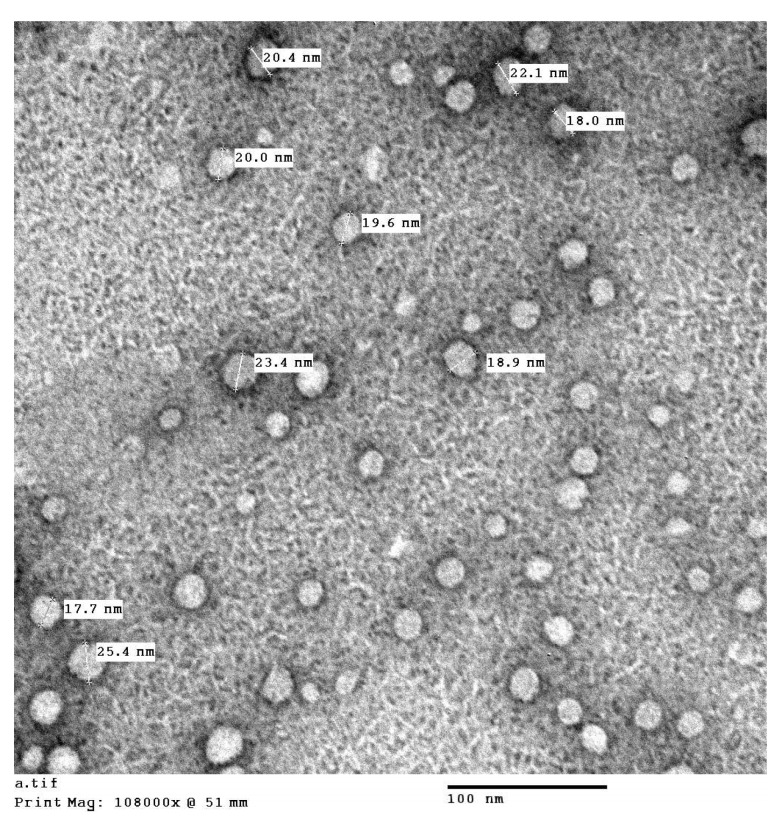
TEM image of 2ME TPGS micelles.

The size of the prepared 2ME-TPGS micelles was assessed and the results revealed vesicle size of 44.3 ± 3.5 nm. In addition, the prepared 2ME-TPGS micelles formula showed 2ME entrapment efficiency % of 62.3 ± 6.7%. The prepared 2ME-TPGS micelles was further characterized by TEM investigation (Figure 1). The TEM results revealed spherical 2ME-TPGS structures with sizes comparable to the vesicle size obtained by the particle size analyzer taking in consideration drying process of the vesicles during TEM sample preparation.

### 3.2. Kidney Function Markers

**Figure 2:**
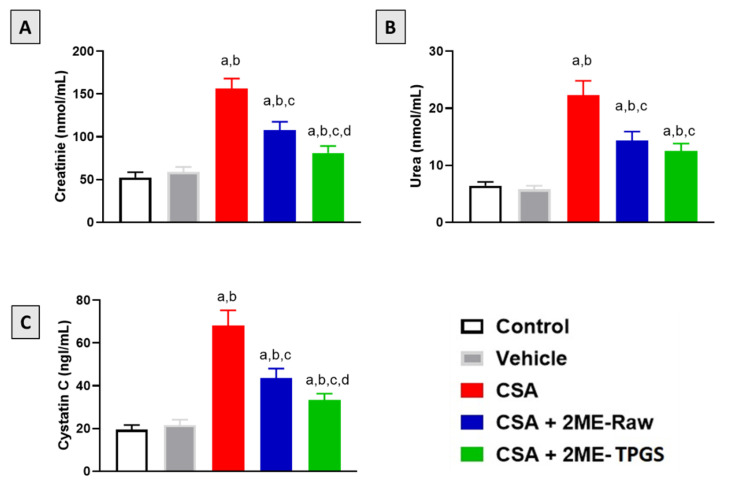
Effect of 2ME-TPGS micelles on serum markers of kidney function following cyclosporine A (CSA) challenge in rats. (**A**): creatinine, **(B**): Urea, (**C**): Cystatin C. Data are presented as Mean ± SD (*n* = 6). a: Significantly different from respective Control at *p* < 0.05; b: significantly different from Vehicle at *p* < 0.05; c: significantly different from CSA at *p* < 0.05; d: significantly different from CSA + 2ME-Raw at *p* < 0.05.

As shown in Figure 2A–C, daily injection of CSA resulted in significant deterioration of kidney function as serum creatinine urea and cystatin C were elevated by approximately 200, 250, and 100% respectively as compared with control values. However, co-treatment of rats with 2ME-Nano significantly prevented the rise of serum creatinine, urea, and cystatin C by 48, 46, and 51%, respectively, as compared with CSA-alone group. Interestingly, 2ME-TPGS micelles ameliorated the rise in serum levels of creatinine and cystatin C when compared with the 2ME-Raw group.

### 3.3. Histopathological Examinations

**Figure 3:**
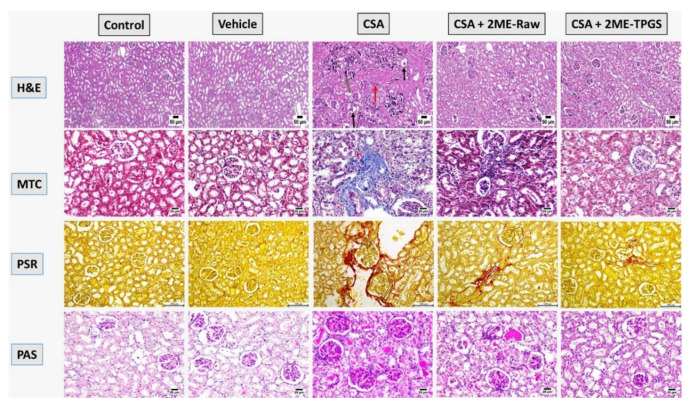
Effects of 2ME-TPGS on CSA-induced kidney injury as visualized using H&E, Masson’s Trichrome (MT), Picrosirius Red (PSR), and Periodic acid–Schiff (PAS) stains. In H&E-stained sections, black arrows indicate atrophy of glomerular capillary tuft and red arrows indicate extensive renal tubular damage. In MT-stained sections, blue coloration indicates collagen deposition. In PSR-stained sections, red coloration indicates collagen deposition. In PAS-stained sections, intense bluish-purple coloration indicates thickening of the glomerular basement membrane.

Microscopic examination of kidneys sections was performed using H&E, MT, PSR, and PAS stains (Figure 3). Sections obtained from control animals revealed absence of any detectable histopathological changes; both renal cortex and medulla were normal. Likewise, kidneys section from the Vehicle-treated group were normal as well. Marked renal damage was detected in CSA-alone treated animals as both renal cortex and medulla were affected. The renal tubular epithelium exhibited marked necrosis with extensive dystrophic calcification represented by the presence of bluish deposits in the necrotized epithelial lining. The glomerular capillary tuft was atrophied in some instances. Some of the examined sections showed cystically dilated tubules while others showed eosinophilic protein-rich cast in their lumens. This was associated with extensive collagen deposition, interstitial fibrosis, and pathological changes in glomerular basement membrane. Moderate improvement was observed in the 2ME-Raw group: some of the renal tubules showed necrosis in their epithelial lining with presence of few calcified spots within the renal cortex. Some renal tubules in the medulla suffered from degeneration and necrosis. Marked alleviation of CSA-induced nephrotoxicity was detected in the 2ME-TPGS micelles group as renal tubules exhibited mild degenerative changes represented by mild cellular swelling, increased cytoplasm granularity, and cellular vacuolation. Some sections of renal cortex were apparently normal. Few sections of renal medulla showed few degenerative changes in tubules meanwhile the rest of examined sections showed apparently normal medulla (Figure 3). Table 2 illustrates a semi-quantitative scoring of histopathological changes considering tubular necrosis, tubular degeneration, tubular dilatation, thickened basement membrane, and interstitial fibrosis. It is obvious that 2ME-TPGS exerted the highest ameliorative activity against CSA-induced tubular pathological changes as well as interstitial fibrosis.

### 3.4. Assessment of Oxidative Status

**Figure 4:**
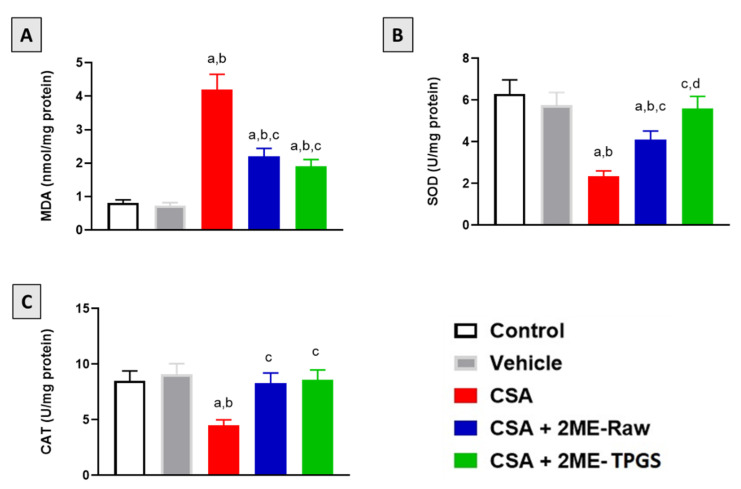
Effect of 2-ME-TPGS on oxidative stress markers in CSA-treated rats. (**A**) MDA; (**B**) SOD; and (**C**) CAT. Data are displayed as mean ± SD (*n* = 6). a: Significantly different from respective Control at *p* < 0.05; b: significantly different from Vehicle at *p* < 0.05; c: significantly different from CSA at *p* < 0.05; d: significantly different from CSA + 2ME-Raw at *p* < 0.05.

As demonstrated in Figure 4A, MDA content in kidney tissue was significantly elevated in the CSA-alone group by 418% as compared with the control group. However, co-treatment of rats with 2ME-Raw and 2ME-Nano significantly inhibited MDA rise by 48% and 55%, respectively, as compared with the CSA-alone group. Furthermore, the data in Figure 4B,C indicate that CSA significantly inhibited SOD and CAT activities in kidney tissue by 62% and 47%, respectively, as compared with the control group. 2ME-Raw significantly prevented exhaustion of SOD and CAT and enhanced their activities by 73% and 84% when compared with the CSA group. Interestingly, SOD and CAT levels were almost completely restored to normal values observed in control and vehicle-treated animals.

### 3.5. Assessment of Inflammation Markers

**Figure 5:**
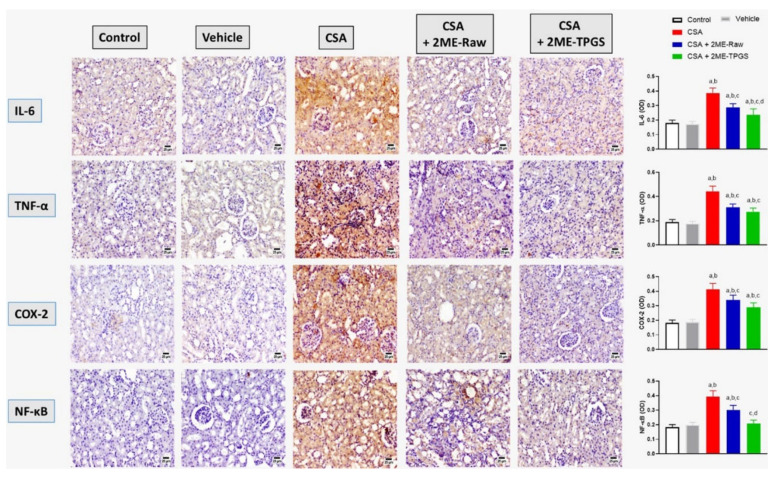
Effect of 2ME-TPGS on IL-6, TNF-α, COX-2 and NFκB expression as determined immunohistochemically in kidney tissues of rats challenged with cyclosporine (CSA). Bar charts are mean ± SD (*n* = 6). a: Significantly different from respective Control at *p* < 0.05; b: significantly different from Vehicle at *p* < 0.05; c: significantly different from CSA at *p* < 0.05; d: significantly different from CSA + 2ME-Raw at *p* < 0.05.

As shown in Figure 5 (upper 2 panels), CSA injection significantly enhanced expression of IL-6 and TNF-α in kidney by 114% and 132%, respectively as compared with the control values. Nevertheless, co-treatment with 2ME-Nano significantly ameliorated these changes by 39% and 37%, respectively, as compared with the corresponding CSA group. A similar pattern was observed when assessing COX-2 expression (3rd panel); CSA injection was associated with 125% increase in COX-2 expression as compared with the control value. Both 2ME-Raw and 2ME-Nano significantly ameliorated such changes and prevented the rise in COX-2 expression by 17% and 30%, respectively, as compared with the CSA group. NF-κB expression was also increased in CSA group (4th panel) by 52% as compared with control group. Yet, 2ME-Raw and 2ME-Nano inhibited such rise by 23% and 47%, respectively, when compared with the CSA group. It is worth mentioning that 2ME-Nano was statistically more protective when compared with 2ME-raw.

### 3.6. mRNA Expression of Bax and Bcl2

**Figure 6:**
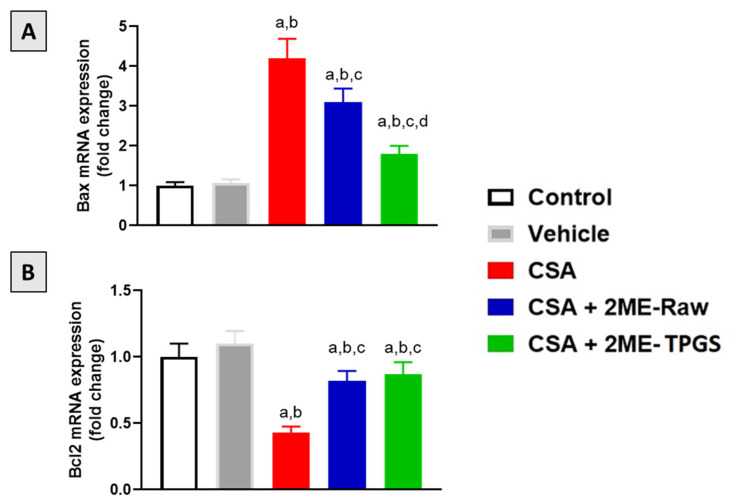
Effect of 2ME-TPGS on kidney mRNA expression of Bax (**A**) and Bcl-2 (**B**) in CSA-treated rats. Data presented in bar charts are expressed as mean ± SD (*n* = 6). a, b, c, or d: significantly different when compared with respective Control, Vehicle, CSA, or CSA + 2ME-Raw at *p* < 0.05, respectively.

The data in Figure 6A demonstrate that mRNA expression of Bax was significantly enhanced in CSA group by 320% as compared with control group. Both 2ME-Raw and 2ME-TPGS prevented such rise by 26% and 57% as compared with CSA group. 2ME-TPGS group was statistically more potent in ameliorating Bax down-regulation. On the other hand, CSA injection resulted in inhibition of Bcl2 mRNA expression by 230% as compared with control. However, 2ME-Raw and 2ME-TPGS ameliorated CSA effects on Bcl2 mRNA expression and exhibited values amounting to 82% and 87% of the control group (Figure 6B).

### 3.7. Western Blot of TGF-β1 and p-ERK1/2

**Figure 7:**
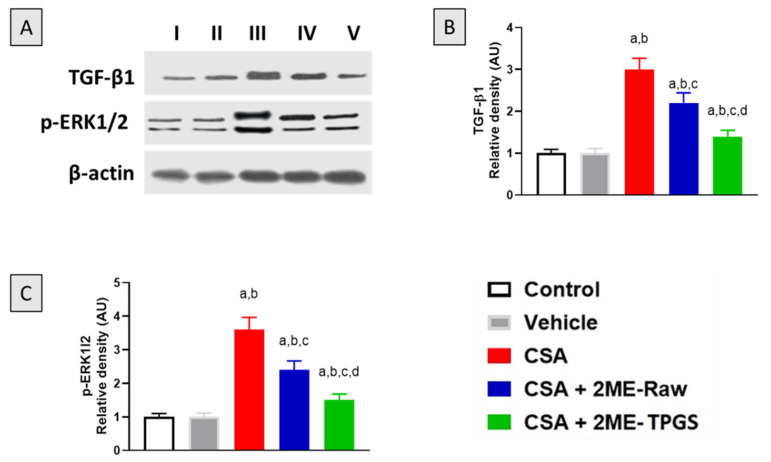
Effect of 2-ME-TPGS on TGF-β1 and phosphorylated ERK1/2 expression in renal tissues of CSA-challenged rats (**A**–**C**). Data in bar charts are shown as mean ± SD (*n* = 6). a, b, c, or d: significantly different when compared with respective Control, Vehicle, CSA, or CSA + 2ME-Raw at *p* < 0.05, respectively.

To further substantiate the protective effects associated with 2-ME-TPGS against CSA-induced nephrotoxicity in rats, protein expression of both TGF-β1 and phosphorylated p-ERK1/2 was assessed. Notably, CSA significantly induced the expression of TGF-β1 by approximately 200% relative to the control values. Yet, 2-ME-Raw and 2ME-TPGS effectively ameliorated the up-regulation of TGF-β1 by 25% and 53%, respectively, as compared with the CSA group (Figure 7A). Similarly, p-ERK1/2 was significantly enhanced in the CSA group by 260% as compared with the control animals. However, 2ME-Raw and 2ME-TPGS prevented p-ERK1/2 by 33% and 58%, respectively, as compared with the CSA group (Figure 7B). One can observe that 2ME-TPGS was statistically superior to 2ME-Raw in attenuating the up-regulation of both TGF-β1 and p-ERK1/2 (Figure 7C).

## 4. Discussion

Nephrotoxicity associated with CSA can be manifested as either acute reversible kidney injury or chronic fibrotic renal dysfunction, and it was even associated with using CSA in the earliest clinical trials using this agent for immunosuppression [53]. CSA-induced nephrotoxicity is progressive and involves permanent lesions associated with tubular atrophy and interstitial fibrosis [54]. Hence, CSA use is restricted because of the high incidence of nephrotoxicity [55,56,57]. 2ME is a metabolite of estrogen and possesses significant protective activities against experimentally-induced renal injury [26,28,29]. However, the therapeutic applications of 2ME are constrained due to its poor solubility which negatively affects its bioavailability and tissue distribution [58,59]. In this regard, loading of 2ME in nanoparticles has been shown to improve its pharmacokinetic and pharmacodynamics characteristics [60,61,62]. Thus, this study aimed at evaluating the potential of 2ME loaded TPGS micelles to prevent CSA-induced nephrotoxicity in rats.

TPGS as a nanocarrier has widely gained attention for its ability to enhance solubility and permeation in various drug delivery systems [45,63,64,65,66,67,68,69,70]. In the current study, the prepared 2ME-TPGS as nanocarrier delivery system for 2ME showed particles size of 44.3 ± 3.5 nm with 2ME entrapment efficiency % of 62.3 ± 6.7%. The size of the prepared TPGS micelles is related to 2ME packing into TPGS micelle core [45,71]. In addition, the improved 2ME entrapment % is attributed to 2ME partitioning in TPGS hydrophobic micelle core at high drug: TPGS ratio which is in agreement with the previous reports [45,72].

Our data indicated that CSA exposure was associated with deterioration of rat kidney functions as indicated by elevated levels of serum creatinine, urea, and cystatin as well as histopathological changes. Interestingly, both 2ME-Raw and 2ME-TPGS exhibited remarkable improvements in kidney functional parameters and histological architecture. These findings are consistent with the reported protective activity of 2-ME in the context ischemia/reperfusion, diabetes, angiotensinII-induced kidney injury in rats [26,27,28,29]. Furthermore, our histopathological indicated that CSA induced collagen deposition and tubulointerstitial fibrosis as shown by different stains. This is consistent with the reported pathological role of fibrosis in CSA-induced renal damage [73]. However, 2ME obviously reduced collagen deposition in this study. Moreover, 2ME loaded TPGS micelles afforded higher degree of protection when compared with raw 2ME. The observed ant-fibrotic effects gain support by several reports highlighting its anti-fibrotic activities of 2ME in the liver [74], lung [75] and kidney [76]. Thus, it can suggest that 2ME anti-fibrotic activity contributes to its protection against CSA-induced kidney injury.

Oxidative stress has been reported to play a major role in CSA-induced nephrotoxicity [77]. This is in line with our findings that showed that CSA-nephrotoxicity was accompanied with oxidative stress. Also, our data indicated that 2ME reno-protective effects against CSA toxicity were mediated by antioxidant effects stress as evidenced by antagonizing MDA accumulation and SOD and CAT exhaustion. These data are in line with the reported antioxidant activities of 2ME [52,78]. In addition, Vitamin E has been previously reported to protect the porcine renal endothelial cell line (LLC-PK1) from mitochondrial damage induced by CSA [79]. Moreover, several molecules protected against CSA nephrotoxicity via mitigating oxidative stress. These include epicatechin [80], quercetin [81], L-carnitine [82] and caffeic acid phenethyl ester [83]. In general, antioxidants have been recommended for prevention of CSA nephrotoxicity [84]. However, the 2-ME-TPGS showed more potent antioxidant activities in comparison with 2ME-Raw. This gains indirect support by the ability of TPGS micelles to enhance the antioxidant activity of hesperetin [85].

A key pathogenic driver in CSA nephrotoxicity is uncontrolled inflammation [73,86,87]. Hence, agents that target this process have been shown to afford protection against CSA nephrotoxicity [49,88,89,90]. In this study, it was observed that a CSA challenge caused increased renal expression of several inflammatory mediators including NF-κB. This is consistent with the reported role of inflammation and NF-κB in CSA renal pathology [91] and might have contributed to the increased expression of IL-6, TNF-α, and COX-2 detected in this study [91]. Nevertheless, 2ME significantly improved the inflammatory status in kidney tissues. This is in harmony with many reports in the literature describing its remarkable inhibitory activity on NF-κB in different cells and tissues [23,25]. These anti-inflammatory effect of 2ME-TPGS were more pronounced when compared with 2ME-Raw. This is consistent with Alhakamy et al. [92]. The decreased expression of these inflammatory cytokines, namely TNF-α and IL-1β, has been reported to reduce apoptosis and pro-apoptotic signaling after kidney injury [27,93]. Consistently, data in the present study indicate that 2-ME significantly ameliorated the rise in mRNA expression of Bax and enhanced Bcl-2 mRNA expression associated with CSA toxicity. This is in line with the reported anti-apoptotic activity of 2ME in kidney tissues [27]. This suggests that the nephroprotection imparted by 2-ME could be also mediated by constraining apoptosis.

To further substantiate the observed protective effects of 2ME, protein expression of TGF-β1 was assessed. Our data indicate that CSA treatment was linked to increased renal TGF-β1 expression. This is consistent with previous studies [82,94,95,96]. 2ME significantly ameliorated CSA-induced up-regulation of TGF-β which has a critical role in renal fibrosis [97]. This is supported by the reported anti-TGF-β1 effect of 2ME in different tissues [52,98]. Other molecules such as fluorofenidone afforded protection against CSA nephrotoxicity via down-regulation of TGF-β expression in kidney tissues [99]. Further, our data indicated that CSA significantly up-regulated protein expression p-ERK1/2. This is supported by previous findings [77]. Yet again, 2ME attenuated activation of ERK1/2. This is in line by a study showing the ability of 2ME to inhibit p-ERK expression in lung tissues [18]. In addition, this is consistent with the reported data linking TGF-β1 to activation of ERK [100] and further highlights the role of both TGF-β1 and p-ERK in the anti-fibrotic activity of 2ME [101]. Interestingly, 2ME-TPGS exhibited more potent activities in suppressing activation of TGF-β1 and p-ERK induced by CSA in rats.

## 5. Conclusions

2ME loaded on nanocarrier protects against CSA-induced kidney injury as evidenced by amelioration of the rise in serum markers of kidney function as well as histopathological changes. This is attributable to 2ME anti-fibrotic, antioxidant, anti-inflammatory, and anti-apoptotic activities which are mediated, at least partly, by inhibition of TGF-β1/p-ERK axis.

## Figures and Tables

**Table 1:** Primer sequences for determination of β-Actin, Bax, and Bcl2 gene expression.

	Forward Primer	Reverse Primer	Accession Number
β-Actin	5′TCCGTCGCCGGTCCACACCC	5′TCACCAACTGGGACGATATG	NM_031144.3
Bax	5′CCTGAGCTGACCTTGGAGCA	5′GGTGGTTGCCCTTTTCTACT	U32098.1
Bcl2	5′TGATAACCGGGAGATCGTGA	5′AAAGCACATCCAATAAAAAGC	NM_016993.1

**Table 2:** Semi-quantitative scoring of tubular injury and fibrosis.

	Tubular Necrosis	Tubular Degeneration	Tubular Dilatation	Thickened Basement Membrane	Interstitial Fibrosis
Control	-	-	-/+	-	-
Vehicle	-	-	-/+	-	-/+
CSA	+++	+++	+++	+++	+++
CSA + 2ME-raw	++	++	+	+	++
CSA + 2ME-TPGS	+	+	+	-/+	+

Non (-), Mild (+), Moderate (++), Severe (+++)

## Data Availability

Data are contained in the article.
